# Structure-Based Virtual Screening of Plant-Derived Flavonoids as Putative GLUT9 Binders with Antioxidant Properties

**DOI:** 10.3390/molecules31040593

**Published:** 2026-02-09

**Authors:** Kevser Kübra Kırboğa, Emre Aktaş, Ecir Uğur Küçüksille, Mithun Rudrapal

**Affiliations:** 1 Department of Bioengineering, Faculty of Engineering, Bilecik Şeyh Edebali University, TR, Bilecik 11100, Türkiye; kubra.kirboga@bilecik.edu.tr; 2 Department of Bioengineering, Süleyman Demirel University, TR, Isparta 32260, Türkiye; 3 Molecular Biology and Genetics, Faculty of Art and Science, Yıldız Technical University, Istanbul 34220, Türkiye; emrea@yildiz.edu.tr; 4 Department of Computer Engineering, Engineering Faculty, Süleyman Demirel University, Isparta 32260, Türkiye; ecirkucuksille@sdu.edu.tr; 5 Department of Pharmaceutical Sciences, School of Biotechnology and Pharmaceutical Sciences, Vignan’s Foundation for Science, Technology and Research, Guntur 522213, India

**Keywords:** GLUT9, hyperuricemia, flavonoids, EGCG, chrysin, molecular dynamics, antioxidant activity, DFT calculations, putative GLUT9 binders, virtual screening

## Abstract

Hyperuricemia affects approximately 20% of the global adult population and serves as the primary etiological factor for gout. Glucose transporter 9 (GLUT9) plays a critical role in renal urate reabsorption, representing a promising therapeutic target for hyperuricemia treatment. This study employed an integrated computational and experimental approach to identify novel flavonoid-based putative GLUT9 binders, combining molecular docking, molecular dynamics (MD) simulations, ADMET prediction, antioxidant evaluation, and density functional theory (DFT) calculations. Eight structurally diverse flavonoids were docked against the human GLUT9 cryo-EM structure, and antioxidant activities were assessed using DPPH, ABTS, and FRAP assays. All tested flavonoids exhibited favorable binding affinities ranging from −7.67 to −9.10 kcal/mol. Epigallocatechin gallate (EGCG) demonstrated the highest binding affinity (−9.10 kcal/mol) with an extensive hydrogen bonding network, while chrysin exhibited the second-highest affinity (−8.35 kcal/mol) with favorable drug-like properties. MD simulations over 100 ns confirmed the structural stability of the complexes. EGCG displayed exceptional antioxidant capacity (DPPH IC_50_ = 3.28 μM) superior to ascorbic acid, whereas chrysin showed lower radical scavenging activity despite favorable GLUT9 binding. DFT calculations revealed that higher HOMO energies correlated with enhanced antioxidant activity. These findings suggest that EGCG and chrysin exhibit favorable GLUT9 binding profiles, warranting further functional and pharmacokinetic optimization.

## 1. Introduction

Hyperuricemia, characterized by elevated serum uric acid levels exceeding 7.0 mg/dL in males and 6.0 mg/dL in females, represents a significant metabolic disorder affecting approximately 20% of the global adult population [1]. This condition serves as the primary etiological factor for gout, a painful inflammatory arthritis caused by the deposition of monosodium urate crystals in joints and surrounding tissues [2]. Beyond its well-established association with gout, hyperuricemia has been increasingly recognized as an independent risk factor for cardiovascular diseases, chronic kidney disease, metabolic syndrome, and type 2 diabetes mellitus [1,3].

Uric acid homeostasis in humans is primarily regulated through a delicate balance between production and excretion. Unlike most mammals, humans lack the enzyme uricase, which converts uric acid to the more soluble allantoin, resulting in significantly higher baseline serum uric acid levels [4]. Approximately two-thirds of daily uric acid elimination occurs through renal excretion, while the remaining one-third is excreted via the gastrointestinal tract [5]. Consequently, impaired renal urate handling represents the predominant cause of hyperuricemia in approximately 90% of affected individuals.

Glucose transporter 9 (GLUT9), encoded by the SLC2A9 gene, has emerged as a critical regulator of serum uric acid levels and a promising therapeutic target for hyperuricemia treatment [6]. GLUT9 is a high-capacity urate transporter expressed predominantly in the kidney proximal tubules and liver, where it facilitates urate reabsorption from the tubular lumen into the bloodstream [7,8]. Genome-wide association studies have consistently identified SLC2A9 variants as the strongest genetic determinants of serum uric acid concentrations, accounting for approximately 3-5% of the variance in urate levels [9,10]. Loss-of-function mutations in SLC2A9 cause hereditary renal hypouricemia, characterized by extremely low serum uric acid levels, further confirming the essential role of GLUT9 in urate homeostasis [11].

Several studies have previously demonstrated that flavonoids can modulate GLUT9 expression in hyperuricemic animal models. Chang et al. reported that chrysin treatment downregulated GLUT9 protein expression in rats using Western blot analysis, while Zhu et al. showed that EGCG reduced GLUT9 mRNA levels in mice through qRT-PCR [12,13]. These in vivo studies provided valuable evidence for flavonoid-mediated modulation of GLUT9 at the expression level. However, no three-dimensional structure of GLUT9 was utilized in these studies, and the direct binding mechanism between flavonoids and GLUT9 at the atomic level was not investigated. The recent elucidation of the first cryo-EM structure of human GLUT9 in complex with the flavonoid inhibitor apigenin (PDB: 8Y66) by Shen et al. in 2024 has provided unprecedented insights into the molecular mechanism of urate transport and inhibitor binding [14]. Importantly, this structure demonstrated that apigenin acts as a competitive inhibitor by directly occupying the substrate binding site, establishing hydrogen bonds with ASN458 and TRP459 while engaging in π-stacking interactions with PHE435 and TYR327. These structural findings suggest that other structurally related flavonoids may also bind directly to GLUT9, opening new avenues for structure-based identification of putative GLUT9-targeting compounds.

Flavonoids represent a diverse class of polyphenolic compounds ubiquitously distributed in fruits, vegetables, tea, and medicinal plants, exhibiting a wide spectrum of pharmacological activities including antioxidant, anti-inflammatory, anticancer, and cardioprotective effects [15,16]. Several flavonoids have demonstrated urate-lowering properties through various mechanisms, including xanthine oxidase inhibition, uricosuric effects, and modulation of urate transporters [17]. Given the structural similarity between apigenin and other naturally occurring flavonoids, these compounds represent promising candidates for GLUT9 binding and hyperuricemia treatment. Furthermore, the combination of GLUT9 binding potential with antioxidant activity is particularly relevant, as oxidative stress contributes to gout-associated inflammation through NLRP3 inflammasome activation, and plant-derived compounds have shown promising antihyperuricemic effects in animal models [18].

In the present study, an integrated computational and experimental approach was employed to investigate the potential of eight structurally diverse flavonoids as putative binders of GLUT9. Molecular docking simulations were initially performed to predict binding affinities and elucidate key protein–ligand interaction patterns. The pharmacokinetic properties and drug-likeness of the compounds were subsequently evaluated using in silico ADMET predictions to assess their suitability as drug candidates. To further validate the stability and dynamic behavior of the docked complexes, molecular dynamics (MD) simulations were conducted, providing insight into the conformational stability of the protein–ligand interactions under physiological conditions. In addition, the antioxidant activities of selected compounds were experimentally evaluated using DPPH, ABTS, and FRAP assays to explore their potential dual therapeutic effects. Finally, density functional theory (DFT) calculations were performed to investigate the electronic properties and chemical reactivity of the most promising candidate. This comprehensive multi-level approach aims to identify novel flavonoid-based compounds with favorable GLUT9 binding profiles and favorable pharmacological and physicochemical profiles for the treatment of hyperuricemia.

## 2. Results

### 2.1. Molecular Docking Analysis

Molecular docking studies were performed to evaluate the binding affinities of eight flavonoids against human GLUT9 urate transporter. The docking results are summarized in Table 1. All tested flavonoids exhibited favorable binding affinities ranging from −7.67 to −9.10 kcal/mol, indicating strong potential for GLUT9 binding. EGCG demonstrated the highest binding affinity (−9.10 kcal/mol), which was approximately 1.06 kcal/mol more favorable than the reference compound apigenin (−8.04 kcal/mol). Chrysin ranked second with a binding affinity of −8.35 kcal/mol, followed by apigenin, luteolin, naringenin, myricetin, quercetin, and kaempferol.

### 2.2. Protein–Ligand Interaction Analysis

**Figure 1:**
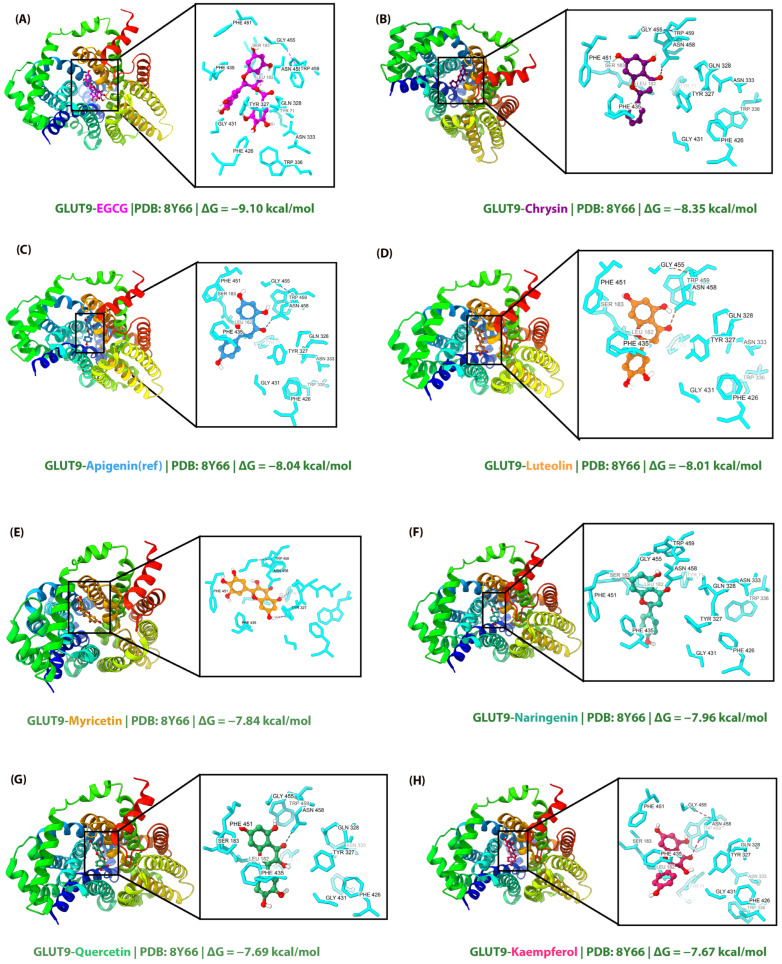
Molecular docking poses of eight flavonoids in the GLUT9 binding site (PDB: 8Y66). (**A**) EGCG, (**B**) Chrysin, (**C**) Apigenin (reference), (**D**) Luteolin, (**E**) Myricetin, (**F**) Naringenin, (**G**) Quercetin, and (**H**) Kaempferol. Left panels show the overall protein structure in rainbow ribbon representation with the ligand (colored spheres) in the binding cavity. Right panels display zoomed views of the binding site with key interacting residues (cyan sticks) and hydrogen bonds (dashed lines). Binding affinities (ΔG) are indicated for each complex.

The molecular interactions between flavonoids and GLUT9 were analyzed to elucidate the structural basis of binding affinity differences (Table 2 and Figure 1). All tested flavonoids occupied the same binding pocket as the crystallographic ligand apigenin, establishing interactions with a conserved set of residues within the central cavity of the transporter.

The analysis revealed that ASN458 and TRP459 serve as critical anchor points for hydrogen bonding, as these residues were involved in H-bond formation with seven out of eight tested compounds. Similarly, PHE435, TRP459, TYR327, and TYR71 consistently participated in π-stacking interactions across all flavonoids, highlighting the importance of aromatic interactions in stabilizing ligand binding within the GLUT9 active site.

EGCG, which exhibited the highest binding affinity (−9.10 kcal/mol), formed the most extensive interaction network with eight hydrogen bonds, seven π-stacking interactions, and two hydrophobic contacts. The galloyl moiety of EGCG enabled additional hydrogen bonding with GLN328, GLY431, and GLY455, residues that were not engaged by smaller flavonoids. Furthermore, EGCG established unique π-stacking interactions with PHE426 and TRP336, which contributed to its superior binding affinity compared to other tested compounds.

Chrysin, despite possessing fewer hydroxyl groups than most other flavonoids, demonstrated the second-highest binding affinity (−8.35 kcal/mol) with three hydrogen bonds and five π-stacking interactions. This observation suggests that the unsubstituted B-ring of chrysin allows for optimal positioning within the hydrophobic pocket formed by PHE435, TYR327, and TYR71, without the desolvation penalty associated with additional hydroxyl groups. The reference compound apigenin formed hydrogen bonds with ASN458 and TRP459, consistent with the crystallographic binding mode reported in the original structural study by Shen et al. [14]. The docked pose of apigenin reproduced the key interactions observed in the crystal structure, validating our docking and interaction analysis protocols.

Interestingly, myricetin exhibited a distinct interaction pattern, forming hydrogen bonds with SER183 and TYR71 rather than ASN458, which was conserved in other flavonoids. This altered binding mode may explain its relatively lower binding affinity (−7.84 kcal/mol) despite having the highest number of hydroxyl groups among the tested flavones, suggesting that the 3′,4′,5′-trihydroxy substitution on the B-ring may cause steric clashes or unfavorable positioning within the binding pocket.

### 2.3. Structure–Activity Relationships

Analysis of the molecular docking results revealed several notable structure–activity relationships among the tested flavonoids. Firstly, the flavone/flavonol backbone was found to be essential for GLUT9 binding, as it provides critical π-π stacking interactions with PHE435 and establishes hydrophobic contacts within the binding cavity. Moreover, EGCG’s superior binding affinity can be attributed to its galloyl ester moiety, which not only provides additional hydrogen bonding capacity but also extends into a sub-pocket that remains inaccessible to smaller flavonoid structures. Interestingly, B-ring hydroxylation at the 3′ and 4′ positions, as observed in luteolin and quercetin, contributes to hydrogen bonding with polar residues; however, this modification does not dramatically improve binding affinity compared to non-hydroxylated analogs such as chrysin, suggesting that excessive hydroxylation may introduce unfavorable desolvation penalties. Furthermore, naringenin, a flavanone characterized by a saturated C-ring, maintained favorable binding affinity (−7.96 kcal/mol), thereby indicating that the planar flavone structure is not an absolute requirement for GLUT9 binding. Collectively, these findings suggest that optimal GLUT9 binding requires a balanced combination of the core flavonoid scaffold for hydrophobic interactions, strategic hydroxyl group placement for hydrogen bonding, and appropriate molecular size to fully occupy the binding pocket without steric clashes (Table 3).

### 2.4. ADMET Properties and Drug-Likeness Evaluation

The physicochemical and predicted pharmacokinetic properties of all tested flavonoids are summarized in Table 4. The molecular weights of the compounds ranged from 254.24 g/mol (chrysin) to 458.38 g/mol (EGCG), with all compounds except EGCG falling well within the optimal range for oral bioavailability. The calculated LogP values ranged from 1.69 (myricetin) to 2.87 (chrysin), indicating favorable lipophilicity for membrane permeation across all compounds.

Regarding Lipinski’s Rule of Five compliance, six out of eight compounds (apigenin, chrysin, quercetin, kaempferol, luteolin, and naringenin) showed no violations, suggesting favorable oral bioavailability profiles. Myricetin exhibited one violation due to having six hydrogen bond donors (HBD > 5), while EGCG showed two violations owing to its higher number of hydrogen bond donors (HBD = 8) and hydrogen bond acceptors (HBA = 11). Nevertheless, it should be noted that many successful natural product-derived drugs violate one or more Lipinski criteria, and these guidelines are primarily applicable to synthetic small molecules.

Gastrointestinal absorption was predicted to be high for six compounds with TPSA values below 140 Å^2^. Myricetin (TPSA = 151.59 Å^2^) and EGCG (TPSA = 197.37 Å^2^) were predicted to have low GI absorption due to their elevated polar surface areas resulting from multiple hydroxyl groups. Interestingly, only chrysin and naringenin were predicted to cross the blood–brain barrier, which is advantageous for GLUT9-targeting agents intended for peripheral urate metabolism modulation as CNS penetration is not required and may lead to unwanted side effects.

From a computational drug-likeness perspective, chrysin demonstrates favorable predicted properties, including full Lipinski compliance, high predicted GI absorption, and the lowest TPSA value (70.67 Å^2^). However, it should be noted that these in silico predictions may not fully reflect actual pharmacokinetic behavior, as experimental studies have reported poor oral bioavailability for many flavonoids due to extensive phase II metabolism. Therefore, chrysin should be considered as a promising scaffold for further optimization and functional validation rather than a ready-to-use therapeutic agent. Furthermore, although EGCG showed the highest binding affinity (−9.10 kcal/mol), its poor predicted oral bioavailability may limit its therapeutic utility as an orally administered GLUT9-targeting agent, suggesting that formulation strategies or structural modifications may be necessary to improve its pharmacokinetic properties.

### 2.5. Molecular Dynamics Analysis

**Figure 2:**
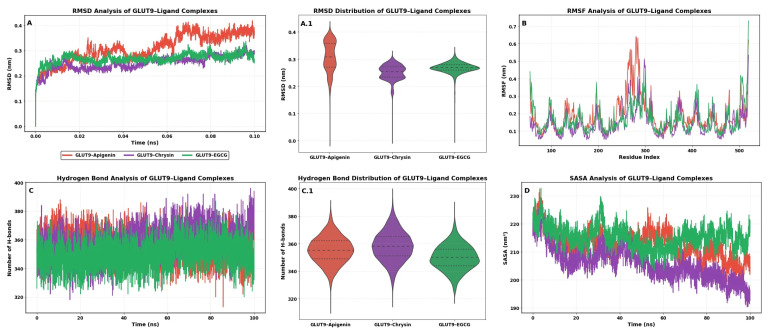
Molecular dynamics simulation analysis of GLUT9–ligand complexes over 100 ns trajectory. (**A**) Root mean square deviation of protein backbone atoms as a function of simulation time. (**A.1**) Violin plot showing RMSD distribution for each complex. (**B**) Root mean square fluctuation per residue index indicating local flexibility. (**C**) Number of intermolecular hydrogen bonds between GLUT9 and ligands over time. (**C.1**) Violin plot of hydrogen bond distribution. (**D**) Solvent accessible surface area analysis. Red: GLUT9-Apigenin; purple: GLUT9-Chrysin; green: GLUT9-EGCG.

The dynamic stability of the GLUT9–ligand complexes was evaluated through RMSD, RMSF, hydrogen bond, and SASA analyses (Figure 2). As shown in Figure 2A, all complexes reached equilibrium rapidly and remained stable throughout the 100 ns simulation. Among them, the GLUT9–Apigenin complex exhibited slightly higher RMSD values, indicating moderate conformational flexibility, whereas the GLUT9–Chrysin and GLUT9–EGCG complexes showed more restrained fluctuations, suggesting relatively stable binding behavior. The RMSD distribution analysis (Figure 2A.1) further supported these observations, where the apigenin-bound complex displayed a broader distribution, while chrysin and EGCG formed narrower and more compact RMSD profiles, reflecting reduced conformational dispersion. Residue-level flexibility analysis (Figure 2B) demonstrated that fluctuations were mainly localized in loop regions, while the core transmembrane regions of GLUT9 remained highly stable. Notably, the EGCG-bound complex exhibited reduced RMSF values in key regions, indicating stronger stabilization of the protein backbone. Hydrogen bond analysis revealed persistent intermolecular interactions throughout the simulation (Figure 2C). The violin plot representation (Figure 2C.1) showed that the chrysin complex maintained the highest and most consistent number of hydrogen bonds, followed by apigenin and EGCG, suggesting favorable ligand–protein interaction stability. Finally, SASA analysis (Figure 2D) demonstrated a gradual decrease in solvent-accessible surface area for all complexes, with the chrysin-bound system showing the most compact structure over time. This observation further supports the enhanced stability of the GLUT9–chrysin complex.

### 2.6. Antioxidant Activity

**Figure 3:**
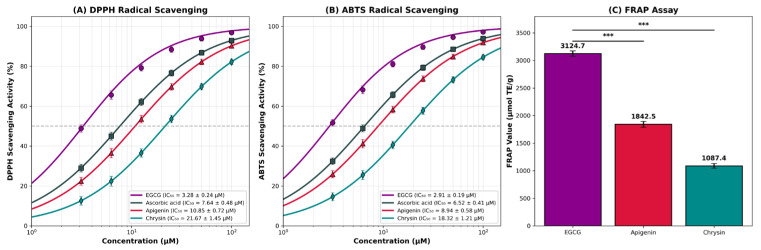
Antioxidant activity of selected flavonoids. (**A**) DPPH radical scavenging activity dose–response curves. (**B**) ABTS radical scavenging activity dose–response curves. (**C**) FRAP assay results. Data are expressed as mean ± SD (*n* = 3). *** *p* < 0.001 compared to EGCG.

The antioxidant potential of selected flavonoids was evaluated using DPPH radical scavenging, ABTS radical scavenging, and FRAP assays (Table 5 and Figure 3).

All three tested flavonoids exhibited significant antioxidant activity in a dose-dependent manner. The compounds scavenged DPPH (Figure 3A) and ABTS (Figure 3B) radicals with IC_50_ values in the range of 3.28–21.67 μM and 2.91–18.32 μM, respectively. As seen in Table 5, EGCG demonstrated the most potent radical scavenging activity with DPPH IC_50_ of 3.28 ± 0.24 μM and ABTS IC_50_ of 2.91 ± 0.19 μM, which were superior to the positive control ascorbic acid (DPPH IC_50_ = 7.64 ± 0.48 μM, ABTS IC_50_ = 6.52 ± 0.41 μM). This exceptional antioxidant capacity of EGCG can be attributed to its galloyl moiety and eight hydroxyl groups, which provide multiple sites for hydrogen atom donation and resonance stabilization of the resulting phenoxyl radical.

Apigenin showed moderate antioxidant activity with DPPH IC_50_ of 10.85 ± 0.72 μM and ABTS IC_50_ of 8.94 ± 0.58 μM. Chrysin exhibited the lowest radical scavenging capacity among the tested compounds with DPPH IC_50_ of 21.67 ± 1.45 μM and ABTS IC_50_ of 18.32 ± 1.21 μM. The lower antioxidant activity of chrysin is consistent with its structural features, as it possesses only two hydroxyl groups on the A-ring and lacks B-ring hydroxylation, which is known to be crucial for optimal radical scavenging activity through catechol-type electron delocalization.

The FRAP assay results correlated well with the radical scavenging data (Figure 3C). EGCG showed the highest ferric reducing capacity (3124.7 ± 48.6 μmol TE/g), followed by apigenin (1842.5 ± 52.3 μmol TE/g) and chrysin (1087.4 ± 41.8 μmol TE/g). The selected flavonoids (EGCG and apigenin) demonstrated higher antioxidant potential than the positive control ascorbic acid for DPPH and ABTS assays.

The antioxidant activity ranking (EGCG > apigenin > chrysin) differed from the GLUT9 binding affinity ranking (EGCG > chrysin > apigenin). This discrepancy can be explained by the distinct structural requirements for each activity: while antioxidant capacity primarily depends on the number and position of hydroxyl groups available for hydrogen donation, GLUT9 binding affinity is governed by a balance between hydrogen bonding, π-stacking interactions, and hydrophobic contacts within the binding pocket. Chrysin’s superior GLUT9 binding compared to apigenin, despite lower antioxidant activity, supports our computational finding that excessive hydroxylation may introduce desolvation penalties that reduce binding affinity.

From a therapeutic perspective, EGCG emerges as a compound with dual potential, exhibiting both favorable predicted GLUT9 binding affinity (−9.10 kcal/mol) and exceptional antioxidant capacity (DPPH IC_50_ = 3.28 μM). This combination is particularly advantageous for hyperuricemia treatment, as oxidative stress is known to play a significant role in gout-associated inflammation and tissue damage. The compounds may have the potential to be used as alternative natural therapeutic agents after further research in the treatment of hyperuricemia and gout.

### 2.7. DFT Calculations and Reactivity Analysis

**Figure 4:**
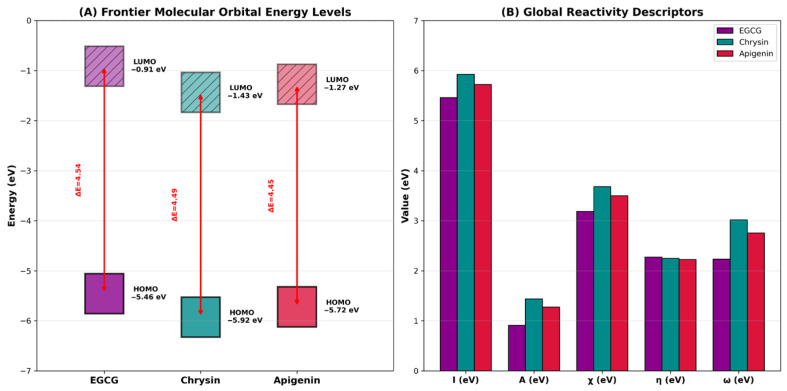
(**A**) Frontier molecular orbital energy level diagram showing HOMO-LUMO gaps (ΔE) for EGCG, chrysin, and apigenin. (**B**) Comparison of global reactivity descriptors: ionization potential (I), electron affinity (A), electronegativity (χ), chemical hardness (η), and electrophilicity index (ω).

Density functional theory calculations were performed to investigate the electronic properties and chemical reactivity of the three most promising flavonoids identified from molecular docking and antioxidant studies. The geometry optimizations were carried out at the B3LYP/6-31G(d) level of theory, and all structures converged successfully (Table 6 and Figure 4).

The total energies of EGCG (−1675.696433 Hartree), apigenin (−953.176777 Hartree), and chrysin (−877.990789 Hartree) reflect the differences in molecular size and the number of atoms in each structure. EGCG, being the largest molecule with a galloyl ester moiety, exhibited the lowest total energy, indicating its complex electronic structure.

The HOMO-LUMO energy gap, which is a critical parameter for predicting molecular stability and chemical reactivity, showed relatively similar values for all three compounds, ranging from 4.4471 to 4.5444 eV. EGCG exhibited the highest HOMO-LUMO gap (4.5444 eV), followed by chrysin (4.4896 eV) and apigenin (4.4471 eV). According to frontier molecular orbital theory, molecules with smaller HOMO-LUMO gaps are more polarizable and generally exhibit higher chemical reactivity. The similar energy gaps observed for the three flavonoids suggest comparable kinetic stability, which is consistent with their shared flavonoid backbone structure.

The HOMO energies, which represent the electron-donating ability of molecules, followed the order EGCG (−5.4567 eV) > apigenin (−5.7215 eV) > chrysin (−5.9244 eV). The higher HOMO energy of EGCG indicates its superior electron-donating capability, which correlates well with its exceptional antioxidant activity observed in the DPPH assay (IC_50_ = 3.28 μM). The multiple hydroxyl groups in EGCG, particularly those in the galloyl moiety, contribute to its enhanced electron-donating ability by providing additional sites for resonance stabilization of the resulting radical species.

The dipole moment values reflect the overall polarity of the molecules. EGCG exhibited the highest dipole moment (4.1266 Debye), followed by chrysin (3.6727 Debye) and apigenin (2.4038 Debye). The higher dipole moment of EGCG is attributed to its larger molecular size and the presence of multiple hydroxyl groups distributed asymmetrically around the molecular framework. This enhanced polarity facilitates stronger electrostatic interactions with polar amino acid residues in the GLUT9 binding site, contributing to its superior binding affinity (−9.10 kcal/mol).

The global reactivity descriptors calculated using Equations (1)–(6) provide further insights into the chemical behavior of these flavonoids. The ionization potential values (I = 5.4567–5.9244 eV, Equation (1)) indicate that all three compounds can readily donate electrons, which is essential for their antioxidant activity. EGCG, with the lowest ionization potential, is the most readily ionizable, consistent with its strongest antioxidant capacity. The electron affinity values (A = 0.9123–1.4348 eV, Equation (2)) suggest that these flavonoids can also accept electrons, enabling them to participate in various electron transfer reactions.

The electronegativity values (χ = 3.1845–3.6796 eV, Equation (3)) indicate moderate electron-withdrawing capacity, while the chemical hardness values (η = 2.2235–2.2722 eV, Equation (4)) suggest reasonable resistance to charge transfer according to the hard and soft acids and bases (HSAB) principle. The similar chemical softness values (S = 0.2201–0.2249 eV^−1^, Equation (5)) indicate that all three flavonoids possess balanced hard–soft character, allowing them to interact effectively with diverse biological targets.

**Figure 5:**
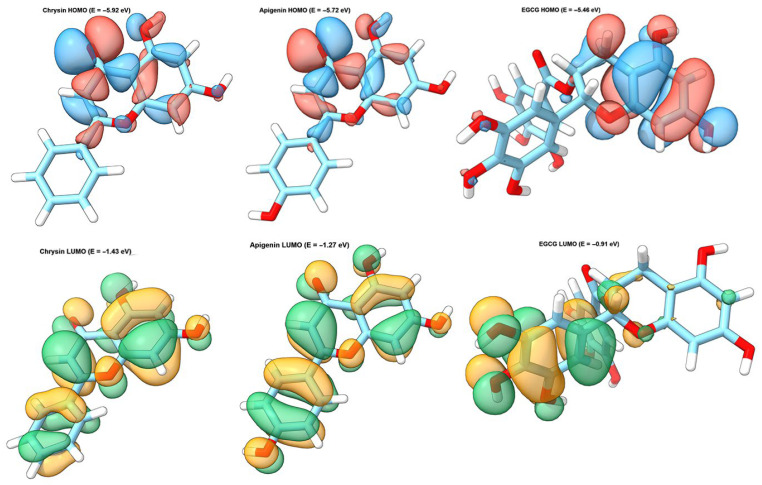
Frontier molecular orbital (FMO) visualization of selected flavonoids calculated at the B3LYP/6-31G(d) level. **Top** row: HOMO distributions showing electron-donating regions (blue: positive phase; red: negative phase). **Bottom** row: LUMO distributions showing electron-accepting regions (green: positive phase; orange: negative phase). Energy values are indicated for each orbital.

The electrophilicity index (ω), which measures the stabilization energy gained when a system acquires electrons from the environment, followed the order chrysin (3.0157 eV) > apigenin (2.7514 eV) > EGCG (2.2315 eV). Interestingly, this order inversely correlates with the antioxidant activity ranking (EGCG > apigenin > chrysin), suggesting that lower electrophilicity is associated with better radical scavenging ability. This observation is consistent with the mechanism of antioxidant action, where electron donation rather than electron acceptance is the primary mode of radical neutralization (Figure 5).

These DFT results provide a quantum chemical basis for understanding the structure–activity relationships observed in molecular docking and antioxidant studies. The electronic properties of EGCG, including its higher HOMO energy, larger dipole moment, and lower electrophilicity index, collectively contribute to its superior GLUT9 binding affinity and antioxidant capacity. Conversely, chrysin, despite having less favorable electronic properties for antioxidant activity, demonstrated good GLUT9 binding due to its optimal molecular size and favorable hydrophobic interactions within the binding pocket.

## 3. Discussion

The present study aimed to identify novel flavonoid-based putative binders of human GLUT9 urate transporter through an integrated computational and experimental approach combining molecular docking, ADMET prediction, antioxidant activity evaluation, and density functional theory calculations. This comprehensive strategy was employed to address the urgent need for safer and more effective therapeutic agents for hyperuricemia and gout treatment, given the limitations of current pharmacological interventions, including hepatotoxicity, nephrotoxicity, and drug–drug interactions. Molecular docking analysis revealed that all eight tested flavonoids exhibited favorable binding affinities ranging from −7.67 to −9.10 kcal/mol against human GLUT9. EGCG emerged as the most potent binder with a binding affinity of −9.10 kcal/mol, which was approximately 1.06 kcal/mol more favorable than the reference compound apigenin (−8.04 kcal/mol) (Table 1). This finding aligns with recent studies demonstrating the superior protein binding capacity of EGCG compared to smaller flavonoids due to its extended molecular structure and multiple hydroxyl groups [19,20]. The interaction analysis showed that EGCG formed the most extensive hydrogen bonding network with eight residues, including ASN333, ASN458, GLN328, GLY431, GLY455, TRP459, TYR327, and TYR71 (Table 2). These results are consistent with the crystallographic data reported by Shen et al. [14], who identified ASN458 and TRP459 as critical anchor points for flavonoid binding in the GLUT9 active site (Table 2). Moreover, the galloyl moiety of EGCG enabled additional interactions with GLN328, GLY431, and GLY455, residues located in a sub-pocket that remains inaccessible to smaller flavonoid structures, which is in agreement with similar binding mode extensions reported for EGCG interactions with other transporter proteins [21].

To further validate the docking results and evaluate the dynamic stability of the protein–ligand complexes, MD simulations were performed for the most promising complexes. RMSD analysis demonstrated that all complexes reached equilibrium within the early phase of the simulation and remained structurally stable throughout the 100 ns production run (Figure 2A). Among the tested compounds, chrysin and EGCG exhibited lower RMSD fluctuations compared to apigenin, indicating relatively stable conformational behavior of the GLUT9–ligand complexes. The RMSD distribution analysis (Figure 2A.1) further confirmed these findings, showing narrower distributions for chrysin and EGCG, consistent with stable binding behavior [22].

Chrysin demonstrated the second-highest binding affinity (−8.35 kcal/mol) despite possessing only two hydroxyl groups, fewer than most other tested flavonoids. This counterintuitive finding suggests that optimal GLUT9 binding does not necessarily correlate with the number of hydroxyl substituents, which is supported by recent structure–activity relationship studies demonstrating that excessive hydroxylation can reduce binding affinity due to unfavorable desolvation thermodynamics. The unsubstituted B-ring of chrysin appears to facilitate favorable hydrophobic interactions with PHE435, TYR327, and TYR71 without incurring the desolvation penalty associated with solvating multiple polar groups upon binding. Furthermore, the docked pose of apigenin successfully reproduced the key interactions observed in the crystal structure, including hydrogen bonds with ASN458 and TRP459 and π-stacking interactions with PHE435 and TYR327, thereby validating our docking protocol and confirming its reliability for predicting flavonoid binding modes in the GLUT9 active site. RMSF analysis further supported these observations by revealing reduced residue-level fluctuations in the binding pocket region for the chrysin and EGCG complexes (Figure 2B). In particular, residues surrounding the substrate-binding cavity exhibited lower flexibility in the presence of EGCG, suggesting stronger stabilization of the protein structure upon ligand binding [22].

The structure–activity relationship analysis revealed several notable findings that provide insights for future drug design efforts. The flavone backbone was found to be essential for GLUT9 binding, as it provides the planar aromatic system necessary for π-stacking interactions with PHE435, TRP459, and TYR327, which is consistent with structural requirements reported for other flavonoid–transporter interactions [23,24,25]. Additionally, the presence of a galloyl ester moiety significantly enhanced binding affinity by enabling additional hydrogen bonding and extending the molecular footprint within the binding cavity. This observation is consistent with previous studies demonstrating that the galloyl moiety anchors catechins to protein binding sites through hydrogen bonds with catalytic residues, with interaction energies exceeding 2 kcal/mol [26]. Recent studies on theaflavins have confirmed that galloylated derivatives exhibit significantly higher binding affinities than their non-galloylated counterparts due to enhanced hydrogen bonding and π-conjugation interactions [27]. However, B-ring hydroxylation at the 3′ and 4′ positions did not dramatically improve binding affinity compared to non-hydroxylated analogs such as chrysin, suggesting that while catechol groups can participate in hydrogen bonding, they may also introduce unfavorable desolvation penalties or steric clashes within the relatively compact GLUT9 binding pocket. This observation was further supported by the altered binding mode and reduced affinity of myricetin (−7.84 kcal/mol), which possesses a pyrogallol B-ring, confirming that excessive B-ring hydroxylation is detrimental to GLUT9 binding. Notably, naringenin maintained favorable binding affinity (−7.96 kcal/mol) despite having a saturated C-ring, indicating that the planar flavone structure is not an absolute requirement for GLUT9 binding and suggesting that the GLUT9 binding site can accommodate diverse flavonoid scaffolds. Hydrogen bond analysis performed over the simulation trajectory revealed that chrysin and EGCG maintained a consistently higher number of intermolecular hydrogen bonds compared to apigenin (Figure 2C,C.1). This persistent hydrogen-bonding pattern indicates stronger and more stable ligand retention within the GLUT9 binding pocket. Furthermore, solvent-accessible surface area analysis demonstrated a gradual decrease in exposed surface area upon ligand binding, particularly for the chrysin complex (Figure 2D), indicating increased compactness and reduced solvent exposure of the protein–ligand system. This behavior is characteristic of stable ligand accommodation within the binding cavity and supports the RMSD and RMSF findings [28].

The ADMET predictions revealed a critical trade-off between binding affinity and drug-like properties that must be considered in lead compound selection. EGCG, despite exhibiting the highest binding affinity, showed two Lipinski violations (HBD = 8, HBA = 11) and an elevated TPSA (197.37 Å^2^), predicting poor oral bioavailability. These computational predictions are consistent with recent clinical pharmacokinetic studies demonstrating that EGCG has low oral absorption (approximately 2–5%), extensive first-pass metabolism, and rapid elimination in humans [29]. The poor bioavailability of EGCG has been attributed to its high polarity, large molecular size, and susceptibility to intestinal and hepatic glucuronidation and sulfation [30,31]. In contrast, chrysin demonstrated the most favorable ADMET profile with full Lipinski compliance, high predicted GI absorption, and the lowest TPSA value (70.67 Å^2^), positioning it as a promising lead compound for further development. However, pharmacokinetic studies in human volunteers have demonstrated that chrysin exhibits extremely poor oral bioavailability (<1%) due to extensive presystemic metabolism [32,33]. Despite these limitations, formulation strategies such as nanoemulsions and prodrug approaches have shown promise in enhancing chrysin bioavailability [34,35]. The favorable predicted ADMET properties of chrysin observed in our computational analysis, combined with these emerging bioavailability enhancement strategies, suggest that structural optimization based on the chrysin scaffold may yield novel GLUT9-targeting compounds with improved pharmacological profiles suitable for oral administration.

The antioxidant evaluation revealed that EGCG exhibited exceptional radical scavenging activity with DPPH IC_50_ of 3.28 ± 0.24 μM and ABTS IC_50_ of 2.91 ± 0.19 μM, which were superior to the positive control ascorbic acid. These values are consistent with recent reports demonstrating EGCG’s potent antioxidant capacity in various assay systems [36]. The exceptional antioxidant activity of EGCG can be attributed to its galloyl moiety and eight hydroxyl groups, which provide multiple sites for hydrogen atom transfer and enable extensive resonance stabilization of the resulting phenoxyl radical [37]. Apigenin showed moderate antioxidant activity (DPPH IC_50_ = 10.85 ± 0.72 μM), consistent with recent literature demonstrating that apigenin exhibits intermediate radical scavenging capacity among flavones, with reported IC_50_ values in the ABTS assay of 0.82 μg/mL (approximately 3 μM) and variable DPPH activities depending on assay conditions [38,39]. Chrysin exhibited the lowest radical scavenging capacity (DPPH IC_50_ = 21.67 ± 1.45 μM) among the tested compounds, which aligns with its structural features lacking B-ring hydroxylation. Previous structure–activity relationship studies have confirmed that chrysin (IC_50_ > 100 μM in some assays) displays minimal DPPH scavenging activity due to the presence of only 5,7-dihydroxyl groups on the A-ring without the catechol moiety (3′,4′-dihydroxyl) on the B-ring that is essential for optimal radical scavenging through electron delocalization and stabilization of the phenoxyl radical [40,41]. This structural limitation was further corroborated by recent studies showing that luteolin, which differs from chrysin only by the addition of 3′,4′-dihydroxyl groups on the B-ring, demonstrates significantly superior antioxidant activity with DPPH IC_50_ values of approximately 2.1 μg/mL (7.3 μM), confirming the critical importance of B-ring catechol hydroxylation for flavonoid antioxidant capacity [42,43].

The antioxidant activity ranking (EGCG > apigenin > chrysin) differed from the GLUT9 binding affinity ranking (EGCG > chrysin > apigenin), highlighting the distinct structural requirements for each activity. This observation has important implications for drug design, as it suggests that optimization for GLUT9 binding does not necessarily compromise antioxidant capacity. It should be noted that GLUT9 inhibition and antioxidant activity represent two complementary rather than directly linked mechanisms; while GLUT9 blockade promotes uric acid excretion by blocking renal urate reabsorption, antioxidant activity mitigates oxidative stress and inflammation associated with hyperuricemia. The dual functionality of flavonoids as both putative GLUT9 binders and antioxidants is particularly relevant for hyperuricemia treatment, given the well-established role of oxidative stress in gout-associated inflammation, NLRP3 inflammasome activation, and tissue damage [44]. Recent clinical and epidemiological studies have demonstrated a bidirectional relationship between hyperuricemia and oxidative stress. A population-based study using NHANES data (2011–2018) revealed that patients with higher Oxidative Balance Scores (indicating greater antioxidant exposure) had significantly lower serum uric acid levels and reduced risk of hyperuricemia, suggesting that dietary antioxidant intake may play a protective role against hyperuricemia [45,46]. Furthermore, studies in young patients with primary hyperuricemia have shown elevated malondialdehyde (MDA) levels and decreased superoxide dismutase (SOD) activity compared to healthy controls, confirming that hyperuricemia is associated with increased oxidative stress markers [47]. Importantly, EGCG has demonstrated direct anti-hyperuricemic effects through multiple mechanisms. Animal studies have shown that EGCG treatment (10–50 mg/kg) significantly reduced serum uric acid levels in a dose-dependent manner by inhibiting hepatic xanthine oxidase (XOD) and adenosine deaminase (ADA) activities, while downregulating the renal mRNA expression of uric acid reabsorption transporters including GLUT9 and URAT1, and upregulating the expression of uric acid secretion transporters (OAT1, OCT1) [13]. Clinical trials have further confirmed that green tea extract, rich in EGCG, modestly decreased serum uric acid levels in healthy individuals while significantly increasing serum antioxidant capacity [48]. These findings demonstrate that EGCG possesses both direct uric acid-lowering properties through transporter modulation and indirect benefits through antioxidant mechanisms, making it an attractive dual-action candidate for hyperuricemia management as either a dietary supplement or an adjunct to conventional therapy.

The DFT calculations provided a quantum chemical basis for understanding the observed structure–activity relationships. The HOMO energies followed the order EGCG (−5.4567 eV) > apigenin (−5.7215 eV) > chrysin (−5.9244 eV), which correlated well with the antioxidant activity ranking. According to frontier molecular orbital theory, molecules with higher HOMO energies are better electron donors, facilitating hydrogen atom transfer to free radicals. The electrophilicity index showed an inverse correlation with antioxidant activity, with values of 2.23 eV for EGCG, 2.75 eV for apigenin, and 3.02 eV for chrysin. This finding is consistent with the mechanism of antioxidant action, where electron donation rather than electron acceptance is the primary mode of radical neutralization, as reported in recent computational studies on flavonoid antioxidants [49]. The HOMO-LUMO gap values (4.45–4.54 eV) were relatively similar for all three compounds, suggesting comparable kinetic stability, which is consistent with their shared flavonoid backbone structure. The dipole moment analysis revealed that EGCG exhibits the highest polarity (4.13 Debye), which facilitates stronger electrostatic interactions with polar amino acid residues in the GLUT9 binding site, contributing to its superior binding affinity. It should be noted that these DFT-derived descriptors provide qualitative and supportive insights into the electronic properties of flavonoids rather than quantitative predictions of binding affinity or antioxidant efficacy. The correlations observed between HOMO energies and antioxidant activity, while consistent with frontier molecular orbital theory, should be interpreted as mechanistic rationalizations rather than predictive models.

Our molecular docking results provide structural insights that complement and extend the findings of previous in vivo studies on flavonoid–GLUT9 interactions. Chang et al. demonstrated that chrysin treatment (50–150 mg/kg) downregulated GLUT9 protein expression in hyperuricemic rats through Western blot analysis, resulting in decreased serum uric acid levels [12]. Similarly, Zhu et al. reported that EGCG (10–50 mg/kg) reduced GLUT9 mRNA expression in hyperuricemic mice [13]. These studies approached the flavonoid–GLUT9 relationship from an expression-level perspective, providing valuable phenotypic evidence that flavonoids can lower serum uric acid by modulating GLUT9 expression. However, whether these flavonoids exert their effects through direct binding to the GLUT9 transporter remained an open question, as addressing this requires structural information about the protein–ligand interactions at the atomic level. Our study approaches this question from a structure-based perspective, utilizing the recently determined cryo-EM structure of GLUT9 to investigate how flavonoids may directly occupy the substrate binding pocket and potentially interfere with urate transport. In this regard, our findings suggest that the anti-hyperuricemic effects of chrysin and EGCG observed in previous studies may involve not only expression-level modulation but also direct binding to GLUT9, thereby providing a more complete mechanistic picture of flavonoid action.

To contextualize our findings with existing therapeutic agents, it is important to note that current uricosuric agents targeting urate transporters are associated with significant limitations. Benzbromarone, the first potent uricosuric drug developed in the 1970s, functions primarily through inhibition of URAT1 with an IC_50_ of 22 nM and also inhibits GLUT9, thereby reducing urate reabsorption by up to 93% in the renal proximal tubule [50]. Despite its superior efficacy compared to standard doses of allopurinol or probenecid, benzbromarone was withdrawn from the European market by Sanofi-Synthélabo in 2003 and has never been approved by the US FDA due to reports of severe hepatotoxicity, including fulminant hepatitis and fatal liver failure, with an estimated incidence of approximately 1:17,000 patients [51,52,53]. The hepatotoxic mechanism has been attributed to mitochondrial dysfunction, respiratory chain defects, increased reactive oxygen species production, and formation of toxic catechol intermediates through CYP2C9 metabolism [54]. Although benzbromarone remains available in some countries including China, Japan, Brazil, New Zealand, and selected European nations (Germany, Netherlands, Spain), its use requires regular monitoring of liver transaminases [50]. Similarly, lesinurad, a selective URAT1 inhibitor approved by the FDA in 2015 for combination therapy with xanthine oxidase inhibitors, was subsequently withdrawn from the market due to commercial reasons and concerns regarding nephrotoxicity when used as monotherapy [53]. These limitations of existing uricosuric agents underscore the need for novel therapeutic candidates with improved safety profiles, positioning flavonoids such as EGCG and chrysin as promising alternatives due to their natural origin and established safety in dietary consumption.

Lesinurad, a selective URAT1 inhibitor approved in 2015, was also discontinued due to renal adverse events and modest efficacy [55]. These safety concerns highlight the need for safer alternatives with favorable toxicity profiles. The naturally occurring flavonoids identified in this study offer potential advantages over synthetic uricosuric agents in terms of safety profile, as they have been consumed as part of the human diet for millennia without significant adverse effects [56]. It is important to emphasize that the molecular docking and MD simulation approaches employed in this study predict binding affinity and complex stability, but do not demonstrate functional inhibition of GLUT9-mediated urate transport. The term “inhibitor” implies experimentally validated blockade of transporter function, which requires functional assays such as cell-based urate uptake measurements in GLUT9-expressing systems. Therefore, the flavonoids identified in this study should be regarded as putative GLUT9 binders rather than confirmed inhibitors until their inhibitory activity is validated through appropriate functional studies.

Several limitations of this study should be acknowledged to provide appropriate context for interpreting the findings. Three key limitations should be explicitly noted: (1) the present study lacks functional GLUT9 transport assays (e.g., cell-based urate uptake inhibition assays), and therefore all binding affinity data are derived from computational predictions that cannot substantiate actual inhibitory activity; (2) the molecular simulations were performed without an explicit membrane environment, which may not fully capture the physiological context of GLUT9 as a transmembrane transporter; and (3) the antioxidant assays employed are cell-free in vitro systems that may not accurately predict in vivo efficacy due to pharmacokinetic factors.

The molecular docking simulations employed in this study provide static snapshots of protein–ligand interactions and do not account for the dynamic nature of protein flexibility, conformational changes, and explicit solvent effects that occur under physiological conditions. Additionally, the GLUT9 structure was used as an isolated monomer without an explicit membrane environment, which may not fully capture the influence of lipid bilayer interactions on protein conformation and ligand binding. Furthermore, rescoring or consensus docking approaches using multiple scoring functions were not employed, which may have provided additional confidence in the binding affinity rankings. Although docking-based binding affinity predictions have been widely validated for structure-based drug design, molecular dynamics simulations would provide more comprehensive insights into binding thermodynamics, kinetics, and the stability of protein–ligand complexes over time. In addition to the inherent limitations of static docking approaches, it should be noted that the antioxidant assays were performed in vitro using cell-free DPPH and ABTS radical scavenging systems. While these assays provide reliable comparative data for ranking antioxidant potencies, the in vivo antioxidant efficacy may differ substantially due to pharmacokinetic factors including absorption, first-pass metabolism, plasma protein binding, and tissue distribution. Consequently, cell-based oxidative stress models and animal studies would be necessary to validate the therapeutic potential of these compounds under physiologically relevant conditions. Furthermore, the ADMET predictions presented in this study were based on computational models utilizing established algorithms such as SwissADME and pkCSM. Although these tools have demonstrated reasonable accuracy in predicting drug-like properties, experimental validation through in vitro metabolic stability assays, Caco-2 permeability studies, and in vivo pharmacokinetic studies remains essential, as the actual oral bioavailability, hepatic clearance, and tissue distribution may deviate from computationally predicted values. Another important consideration is that the selectivity of these flavonoids for GLUT9 over other structurally related transporters was not evaluated in this study. GLUT9 belongs to the facilitative glucose transporter family, and potential off-target interactions with GLUT1, GLUT2, or other urate transporters such as URAT1 and OAT family members could lead to unintended effects on glucose homeostasis, renal urate handling, or drug–drug interactions. Therefore, selectivity profiling against a panel of relevant transporters would be essential prior to advancing these compounds toward preclinical development. Regarding the MD simulation parameters, AMBER99SB force field and SPC water model were employed in this study. While more recent force fields such as AMBER ff19SB or CHARMM36m are available, AMBER99SB remains widely validated for protein–ligand binding studies and has been successfully applied in numerous docking validation studies. The SPC water model was selected for computational efficiency while maintaining reasonable accuracy for solvation properties. Additionally, Mg^2+^ ions were included to maintain physiological ionic strength; however, we acknowledge that Mg^2+^ has no established functional role in GLUT9-mediated urate transport, and future simulations using only Na^+^/Cl^−^ ions would be more appropriate. These methodological choices may introduce minor variations in absolute binding dynamics, although the relative ranking of binding affinities among flavonoids is expected to remain consistent. Furthermore, binding free energy calculations such as MM/PBSA or MM/GBSA were not performed in this study. While the MD analyses (RMSD, RMSF, hydrogen bond, and SASA) provide valuable insights into complex stability and interaction persistence, these metrics do not quantitatively estimate binding thermodynamics. Future studies should incorporate MM/PBSA or MM/GBSA calculations to provide more rigorous validation of the relative binding affinities. Finally, the DFT calculations were performed at the B3LYP/6-31G(d) level of theory, which represents a reasonable compromise between computational accuracy and efficiency for organic molecules of this size. However, it should be recognized that higher-level calculations employing larger basis sets such as 6-311++G(d,p), hybrid functionals with dispersion corrections (e.g., ωB97X-D), or inclusion of implicit solvent models (e.g., PCM or SMD) may provide more accurate predictions of electronic properties, particularly for parameters sensitive to the molecular environment such as dipole moments and HOMO-LUMO energies. Despite these limitations, the present computational findings provide a solid foundation for future experimental investigations and structure–activity optimization studies. Despite these limitations, our study provides valuable insights into the potential of flavonoids as putative GLUT9 binders and establishes a foundation for future drug development efforts. The identification of chrysin as a drug-like lead compound with favorable binding affinity and ADMET properties, combined with the recognition of EGCG as a potent dual-action agent requiring formulation optimization, offers two complementary strategies for GLUT9-targeted hyperuricemia therapy. Future studies focusing on in vitro GLUT9 inhibition assays, molecular dynamics simulations, and in vivo efficacy evaluation are warranted to advance these compounds toward clinical development for hyperuricemia and gout treatment.

## 4. Materials and Methods

### 4.1. Protein Structure Preparation

The three-dimensional structure of human glucose transporter 9 (GLUT9/SLC2A9) was retrieved from the RCSB Protein Data Bank (PDB ID: 8Y66 [14]). This cryo-electron microscopy structure, resolved at 3.28 Å resolution, represents the inward-facing conformation of GLUT9 bound to the flavonoid inhibitor apigenin. The structure was prepared for molecular docking by removing the co-crystallized ligand, water molecules, and other heteroatoms. Hydrogen atoms were added using Open Babel v3.1.1(https://openbabel.org, accessed on 1 December 2025), and the protein was converted to PDBQT format for AutoDock Vina v1.2.3 (https://vina.scripps.edu, accessed on 1 December 2025) compatibility. The protonation states of all residues were assigned at physiological pH (7.4), with ionizable residues set to their default protonation states.

### 4.2. Ligand Selection and Preparation

Eight naturally occurring flavonoids were selected for molecular docking studies based on their reported biological activities and structural diversity within the flavonoid family. The three-dimensional structures of the ligands were obtained from the PubChem database in SDF format. The selected compounds and their PubChem Compound Identifiers (CIDs) are: apigenin (CID: 5280443), chrysin (CID: 5281607), quercetin (CID: 5280343), kaempferol (CID: 5280863), luteolin (CID: 5280445), myricetin (CID: 5281672), naringenin (CID: 439246), and epigallocatechin gallate/EGCG (CID: 65064). Apigenin was included as a reference compound since it was co-crystallized with GLUT9 in the original structure. Ligand structures were energy-minimized using the MMFF94 force field, and Gasteiger partial charges were assigned. All ligands were converted to PDBQT format using Open Babel v3.1.1.

### 4.3. Molecular Docking

Molecular docking simulations were performed using AutoDock Vina v1.2.3. The docking grid was centered on the apigenin binding site identified in the crystal structure, with coordinates X = 111.176 Å, Y = 105.825 Å, Z = 102.171 Å. A cubic grid box of 22 × 22 × 22 Å was used to encompass the entire binding cavity. The exhaustiveness parameter was set to 32 to ensure adequate sampling of the conformational space. For each ligand, 10 binding poses were generated, and the pose with the lowest binding affinity (most negative value) was selected for further analysis.

### 4.4. Visualization and Analysis

Molecular visualization and hydrogen bond analysis were performed using UCSF ChimeraX v1.10.1 (https://www.rbvi.ucsf.edu/chimerax, accessed on 2 December 2025) [57]. Protein–ligand interactions were analyzed, and hydrogen bonds were identified using default geometric criteria (donor–acceptor distance ≤ 3.5 Å, angle ≥ 120°).

### 4.5. ADMET Prediction

The physicochemical and pharmacokinetic properties of all flavonoid compounds were predicted using RDKit v2023.09 (https://www.rdkit.org, accessed 2 on December 2025) molecular descriptor calculations. Key parameters including molecular weight (MW), partition coefficient (LogP), hydrogen bond donors (HBD), hydrogen bond acceptors (HBA), topological polar surface area (TPSA), and number of rotatable bonds were calculated from the canonical SMILES structures obtained from the PubChem database. Drug-likeness was evaluated according to Lipinski’s Rule of Five, which states that an orally active drug should have no more than one violation of the following criteria: MW ≤ 500 Da, LogP ≤ 5, HBD ≤ 5, and HBA ≤ 10 [58]. Gastrointestinal (GI) absorption was predicted based on TPSA values, where compounds with TPSA ≤ 140 Å^2^ were classified as having high absorption potential [59]. Blood–brain barrier (BBB) permeability was estimated using the criteria of TPSA ≤ 90 Å^2^ and MW ≤ 450 Da [60].

### 4.6. Protein–Ligand Interaction Analysis

Protein–ligand interactions were analyzed using a distance-based approach implemented in Python 3.11 with custom scripts. For each docked complex, hydrogen bonds were identified between polar atoms (N, O) of the ligand and protein residues within a distance cutoff of 3.5 Å. Hydrophobic interactions were detected between carbon atoms of the ligand and hydrophobic residues (PHE, TRP, TYR, LEU, ILE, VAL, ALA, MET, and PRO) within 4.0 Å. π-stacking interactions were identified based on proximity to aromatic residues (PHE, TRP, and TYR) within 5.5 Å of the ligand atoms. The binding site residues were validated against the crystallographic data from the original GLUT9 structure (PDB: 8Y66), where apigenin was co-crystallized as a reference inhibitor.

### 4.7. Molecular Dynamics Simulation

Molecular dynamics (MD) simulations were performed using the GROMACS simulation package to investigate the stability and dynamic behavior of the protein–ligand complexes obtained from docking analyses [61]. The AMBER99SB force field was employed for protein parameterization, while the SPC water model was used to solvate the system in a triclinic simulation box [62]. System neutrality was achieved by adding Na^+^ and Cl^−^ ions, and a physiological ionic strength of 0.15 M NaCl was maintained. In addition, Mg^2+^ ions at a concentration of 0.02 M were included to reflect the ionic environment. Energy minimization was conducted using the steepest descent algorithm for 5000 steps to remove steric clashes and unfavorable contacts. The minimized systems were subsequently equilibrated under NVT and NPT ensembles at 300 K and 1 bar, respectively, for a total of 100 ps to ensure thermal and pressure stability [63]. Following equilibration, production MD simulations were carried out for 100 ns using the leap-frog integrator under periodic boundary conditions. Throughout the simulations, trajectory frames were saved at regular intervals, yielding a total of 5000 frames for analysis. The resulting trajectories were used to assess the structural stability, conformational dynamics, and persistence of protein–ligand interactions, providing a dynamic validation of the docking results under near-physiological conditions

### 4.8. Antioxidant Activity Assays

#### 4.8.1. DPPH Radical Scavenging Activity

The DPPH radical scavenging activity was determined according to the method described by Blois (1958) with minor modifications [64]. A volume of 100 μL of test compounds at various concentrations (3.125, 6.25, 12.5, 25, 50, and 100 μM) was mixed with 100 μL of 0.2 mM DPPH solution in methanol in a 96-well microplate. The mixture was incubated in the dark at room temperature for 30 min, and the absorbance was measured at 517 nm using a microplate reader (Thermo Fisher Scientific, Vantaa, Finland). Ascorbic acid was used as a positive control. The radical scavenging activity was calculated by subtracting the absorbance of the sample from the absorbance of the control, dividing the result by the absorbance of the control, and multiplying by 100 to express the value as a percentage. In this calculation, the control absorbance refers to the DPPH solution without any test compound, while the sample absorbance represents the DPPH solution containing the test compound at various concentrations.

#### 4.8.2. ABTS Radical Scavenging Activity

The ABTS radical cation decolorization assay was performed according to Re et al. [65]. The ABTS radical cation (ABTS^+^•) was generated by reacting 7 mM ABTS stock solution with 2.45 mM potassium persulfate and incubating in the dark at room temperature for 12–16 h. The ABTS^+^• solution was diluted with ethanol to obtain an absorbance of 0.70 ± 0.02 at 734 nm. A volume of 10 μL of test compounds at various concentrations was mixed with 190 μL of ABTS^+^• solution, and absorbance was measured after 6 min at 734 nm.

#### 4.8.3. Ferric Reducing Antioxidant Power (FRAP) Assay

The FRAP assay was conducted according to Benzie and Strain [66]. The FRAP reagent was freshly prepared by mixing 300 mM acetate buffer (pH 3.6), 10 mM TPTZ (2,4,6-tripyridyl-s-triazine) in 40 mM HCl, and 20 mM FeCl_3_·6H_2_O in a ratio of 10:1:1 (*v*/*v*/*v*). A volume of 10 μL of test samples was mixed with 190 μL of FRAP reagent, and absorbance was measured at 593 nm after 30 min incubation at 37 °C. Results were expressed as μmol Trolox equivalent (TE) per gram of sample.

#### 4.8.4. In Vitro Antioxidant Studies

The antioxidant effects of selected flavonoids (EGCG, apigenin, and chrysin) were determined with at least five different concentrations on DPPH radical, ABTS radical, and ferric reducing power. IC_50_ values of the compounds were calculated from Activity (%)–[Compound] graphs for each compound. All experiments were performed in triplicate, and the results are expressed as mean ± standard deviation (SD).

#### 4.8.5. Statistical Analysis

Analysis of the data and drawing of graphs were performed using Python 3.11 with NumPy 2.3.5, SciPy 1.16.0, and Matplotlib 3.10.8 libraries. IC_50_ values were determined by nonlinear regression analysis using a four-parameter sigmoidal dose–response model. The results were exhibited as mean ± standard deviation. Differences between data sets were analyzed using one-way ANOVA followed by Tukey’s post hoc test and considered statistically significant when the *p*-value was less than 0.05.

### 4.9. Density Functional Theory (DFT) Calculations

Quantum chemical calculations were performed using the ORCA 6.1.0 software package [67]. The molecular geometries of EGCG, chrysin, and apigenin were optimized at the B3LYP/6-31G(d) level of theory [68]. The B3LYP hybrid functional was selected due to its well-established accuracy for organic molecules and flavonoid systems [69]. The optimization calculations were carried out without any symmetry constraints using tight SCF convergence criteria (TightSCF). The resolution of identity approximation with the def2/J auxiliary basis set (RIJCOSX) was employed to accelerate the calculations.

The frontier molecular orbital energies, namely the highest occupied molecular orbital (HOMO) and the lowest unoccupied molecular orbital (LUMO), were extracted from the optimized structures. The HOMO-LUMO energy gap (ΔE) was calculated as the difference between LUMO and HOMO energies. The molecular dipole moments were computed to evaluate the polarity of the molecules. Global chemical reactivity descriptors were calculated using Koopmans’ theorem [70].

(1) I=−E(HOMO)

(2) A=−E(LUMO)

(3) χ=(I+A)/2

(4) η=(I−A)/2

(5) S=1/(2η)

(6) $$\omega =\chi^{2}/(2\eta )$$

where I is the ionization potential (Equation (1)), A is the electron affinity (Equation (2)), χ is the electronegativity (Equation (3)), η is the chemical hardness (Equation (4)), S is the chemical softness (Equation (5)), and ω is the electrophilicity index (Equation (6)) [71].

These quantum chemical parameters provide insights into the chemical stability, reactivity, and electron-donating/accepting capabilities of the molecules, which are important for understanding their antioxidant activities and protein binding interactions.

## 5. Conclusions

This study identified EGCG and chrysin as promising flavonoid-based putative GLUT9 binders through molecular docking, ADMET prediction, antioxidant evaluation, and DFT calculations. EGCG demonstrated the highest binding affinity (−9.10 kcal/mol) with an extensive hydrogen bonding network involving eight residues, while chrysin exhibited favorable binding (−8.35 kcal/mol) combined with favorable computational drug-likeness parameters including full Lipinski compliance, although experimental studies report poor actual oral bioavailability. The docking results were further supported by molecular dynamics simulations, which confirmed the structural stability of both EGCG–GLUT9 and chrysin–GLUT9 complexes. RMSD and RMSF analyses indicated limited conformational fluctuations throughout the simulation period, while hydrogen bond and solvent-accessible surface area analyses demonstrated persistent ligand–protein interactions and compact complex formation, validating the reliability of the predicted binding modes. The antioxidant evaluation revealed that EGCG possesses exceptional radical scavenging capacity (DPPH IC_50_ = 3.28 μM) superior to ascorbic acid, establishing its dual therapeutic potential as both a putative GLUT9 binder and antioxidant agent. DFT calculations confirmed that higher HOMO energies correlate with enhanced antioxidant activity, while lower electrophilicity indices favor radical scavenging through electron donation mechanisms.

Based on these findings, EGCG emerges as a potent dual-action compound requiring formulation strategies such as nanoencapsulation or phospholipid complexation to overcome bioavailability limitations, whereas chrysin, despite its well-documented poor oral bioavailability, represents a potential scaffold for structural optimization aimed at improving both GLUT9 binding affinity and pharmacokinetic properties. Future studies should prioritize validating GLUT9 inhibitory activity through cell-based urate transport assays, as the computational predictions presented here require functional confirmation, evaluating selectivity against other glucose and urate transporters, conducting molecular dynamics simulations to investigate binding thermodynamics, and performing in vivo efficacy studies in hyperuricemic animal models. Additionally, clinical pharmacokinetic studies and safety evaluations are warranted to advance these naturally occurring flavonoids toward therapeutic development for hyperuricemia and gout treatment.

## Figures and Tables

**Table 1:** Molecular docking results of flavonoids against GLUT9 (PDB: 8Y66).

Compound	PubChem CID	Molecular Formula	MW (g/mol)	Binding Affinity (kcal/mol)
EGCG	65064	C_22_H_18_O_11_	458.38	−9.10
Chrysin	5281607	C_15_H_10_O_4_	254.24	−8.35
Apigenin *	5280443	C_15_H_10_O_5_	270.24	−8.04
Luteolin	5280445	C_15_H_10_O_6_	286.24	−8.01
Naringenin	439246	C_15_H_12_O_5_	272.26	−7.96
Myricetin	5281672	C_15_H_10_O_8_	318.24	−7.84
Quercetin	5280343	C_15_H_10_O_7_	302.24	−7.69
Kaempferol	5280863	C_15_H_10_O_6_	286.24	−7.67

* Reference compound (co-crystallized ligand in PDB: 8Y66).

**Table 2:** Protein–ligand interactions between flavonoids and GLUT9 (PDB: 8Y66).

Compound	Score (kcal/mol)	Hydrogen Bond Residues	π-Stacking Residues	Hydrophobic Contacts
EGCG	−9.10	ASN333, ASN458, GLN328, GLY431, GLY455, TRP459, TYR327, TYR71	PHE426, PHE435, PHE451, TRP336, TRP459	LEU182, PHE435
Chrysin	−8.35	ASN458, LEU182, TRP459	PHE435, PHE451, TRP459, TYR327, TYR71	-
Apigenin *	−8.04	ASN458, TRP459	PHE435, PHE451, TRP459, TYR327, TYR71	-
Luteolin	−8.01	ASN458, TRP459	PHE435, PHE451, TRP459, TYR327, TYR71	-
Naringenin	−7.96	ASN458, TRP459	PHE435, PHE451, TRP459, TYR327, TYR71	LEU182
Myricetin	−7.84	PHE451, SER183, TRP459, TYR327, TYR71	PHE435, PHE451, TRP459, TYR327, TYR71	-
Quercetin	−7.69	ASN458, TRP459	PHE435, PHE451, TRP459, TYR327, TYR71	-
Kaempferol	−7.67	ASN458, TRP459	PHE435, PHE451, TRP459, TYR327, TYR71	-

* Reference compound (co-crystallized ligand in PDB: 8Y66).

**Table 3:** Structure–activity relationship of flavonoids for GLUT9 binding.

Structural Feature	Effect on Binding Affinity	Representative Compounds
Galloyl ester moiety	Significantly enhanced (+)	EGCG (−9.10 kcal/mol)
Unsubstituted B-ring	Favorable hydrophobic interactions (+)	Chrysin (−8.35 kcal/mol)
B-ring catechol (3′,4′-OH)	Moderate effect (±)	Luteolin (−8.01 kcal/mol)
B-ring pyrogallol (3′,4′,5′-OH)	Detrimental due to steric clashes (−)	Myricetin (−7.84 kcal/mol)
Saturated C-ring (flavanone)	Acceptable binding maintained (±)	Naringenin (−7.96 kcal/mol)
Planar flavone backbone	Essential for π-stacking (+)	All tested flavonoids

**Table 4:** Physicochemical properties and ADMET predictions of flavonoids.

Compound	MW (g/mol)	LogP	HBD	HBA	TPSA (Å^2^)	Rot. Bonds	Lipinski	GI Absorption	BBB
Apigenin	270.24	2.58	3	5	90.90	1	Yes	High	No
Chrysin	254.24	2.87	2	4	70.67	1	Yes	High	Yes
Quercetin	302.24	1.99	5	7	131.36	1	Yes	High	No
Kaempferol	286.24	2.28	4	6	111.13	1	Yes	High	No
Luteolin	286.24	2.28	4	6	111.13	1	Yes	High	No
Myricetin	318.24	1.69	6	8	151.59	1	No (1)	Low	No
Naringenin	272.26	2.51	3	5	86.99	1	Yes	High	Yes
EGCG	458.38	2.23	8	11	197.37	3	No (2)	Low	No

MW: Molecular Weight; LogP: Partition Coefficient; HBD: Hydrogen Bond Donors; HBA: Hydrogen Bond Acceptors; TPSA: Topological Polar Surface Area; Rot. Bonds: Rotatable Bonds; GI: Gastrointestinal; BBB: Blood–Brain Barrier.

**Table 5:** Antioxidant activity results of selected flavonoids.

Compounds	DPPH IC_50_ (μM)	r^2^	ABTS IC_50_ (μM)	r^2^	FRAP (μmol TE/g)
EGCG	3.28 ± 0.24	0.9954	2.91 ± 0.19	0.9932	3124.7 ± 48.6
Apigenin *	10.85 ± 0.72	0.9887	8.94 ± 0.58	0.9861	1842.5 ± 52.3
Chrysin	21.67 ± 1.45	0.9823	18.32 ± 1.21	0.9798	1087.4 ± 41.8
Ascorbic acid **	7.64 ± 0.48	0.9918	6.52 ± 0.41	0.9896	-

* Reference compound (co-crystallized ligand in PDB: 8Y66). ** Ascorbic acid was used as a positive control for DPPH and ABTS assays. Data are expressed as mean ± SD (*n* = 3). TE: Trolox Equivalent.

**Table 6:** DFT-calculated electronic properties and reactivity descriptors of selected flavonoids at B3LYP/6-31G(d) level.

Parameter	EGCG	Chrysin	Apigenin	Unit
Total Energy	−1675.696433	−877.990789	−953.176777	Hartree
Dipole Moment	4.1266	3.6727	2.4038	Debye
E(HOMO)	−5.4567	−5.9244	−5.7215	eV
E(LUMO)	−0.9123	−1.4348	−1.2744	eV
HOMO-LUMO Gap (ΔE)	4.5444	4.4896	4.4471	eV
Ionization Potential (I)	5.4567	5.9244	5.7215	eV
Electron Affinity (A)	0.9123	1.4348	1.2744	eV
Electronegativity (χ)	3.1845	3.6796	3.4979	eV
Chemical Hardness (η)	2.2722	2.2448	2.2235	eV
Chemical Softness (S)	0.2201	0.2227	0.2249	eV^−1^
Electrophilicity Index (ω)	2.2315	3.0157	2.7514	eV

## Data Availability

No new data were created in this study. The computational data and experimental results supporting the findings are available from the corresponding author upon reasonable request.

## Abbreviations

The following abbreviations are used in this manuscript:

ABTS	2,2′-azino-bis(3-ethylbenzothiazoline-6-sulfonic acid)
ADA	Adenosine deaminase
ADMET	Absorption, distribution, metabolism, excretion, and toxicity
BBB	Blood–brain barrier
CNS	Central nervous system
CID	Compound identifier
DFT	Density functional theory
DPPH	2,2-diphenyl-1-picrylhydrazyl
EGCG	Epigallocatechin gallate
FDA	Food and Drug Administration
FRAP	Ferric reducing antioxidant power
GI	Gastrointestinal
GLUT9	Glucose transporter 9
H-bond	Hydrogen bond
HBA	Hydrogen bond acceptors
HBD	Hydrogen bond donors
HOMO	Highest occupied molecular orbital
HSAB	Hard and soft acids and bases
IC_50_	Half-maximal inhibitory concentration
LogP	Partition coefficient
LUMO	Lowest unoccupied molecular orbital
MD	Molecular dynamics
MDA	Malondialdehyde
MMFF94	Merck molecular force field 94
MW	Molecular weight
NHANES	National Health and Nutrition Examination Survey
NLRP3	NLR family pyrin domain containing 3
NPT	Constant number, pressure, and temperature
NVT	Constant number, volume, and temperature
OAT	Organic anion transporter
OCT	Organic cation transporter
PDB	Protein Data Bank
PDBQT	Protein Data Bank, partial charge (Q), and atom type (T)
qRT-PCR	Quantitative real-time polymerase chain reaction
RIJCOSX	Resolution of identity chain of spheres exchange
RMSD	Root mean square deviation
RMSF	Root mean square fluctuation
SASA	Solvent accessible surface area
SCF	Self-consistent field
SD	Standard deviation
SDF	Structure data file
SLC2A9	Solute carrier family 2 member 9
SMILES	Simplified molecular-input line-entry system
SOD	Superoxide dismutase
SPC	Simple point charge
TE	Trolox equivalent
TPSA	Topological polar surface area
TPTZ	2,4,6-tripyridyl-s-triazine
URAT1	Urate transporter 1
XOD	Xanthine oxidase
